# Temporal trends of all-cause mortality in Paget’s disease of bone: a population-based retrospective cohort study over 20 yr

**DOI:** 10.1093/jbmrpl/ziaf175

**Published:** 2025-11-05

**Authors:** Laetitia Michou, Philippe Gamache, Louis Rochette, Isaora Zefania Dialahy, Jason R Guertin, Jean-Eric Tarride, Jacques P Brown, Sonia Jean

**Affiliations:** CHU de Québec-Université Laval Research Centre, Endocrionology-nephrology, Population Health and Best Practices in Health and Immunology-infectiology axes, Québec, QC, G1V 4G2, Canada; Department of Medicine, Université Laval, Québec, QC, G1V 0A6, Canada; Institut National de Santé Publique du Québec, Evaluation of programs and surveillance of chronic diseases, injuries and their determinants, Statistical expertise and Information Centre, Québec, QC, G1V 5B3, Canada; Institut National de Santé Publique du Québec, Evaluation of programs and surveillance of chronic diseases, injuries and their determinants, Statistical expertise and Information Centre, Québec, QC, G1V 5B3, Canada; Institut National de Santé Publique du Québec, Evaluation of programs and surveillance of chronic diseases, injuries and their determinants, Statistical expertise and Information Centre, Québec, QC, G1V 5B3, Canada; CHU de Québec-Université Laval Research Centre, Endocrionology-nephrology, Population Health and Best Practices in Health and Immunology-infectiology axes, Québec, QC, G1V 4G2, Canada; Department of Preventive and Social Medicine, Université Laval, Québec, QC, G1V 0A6, Canada; Center of Research in Experimental Organogenesis of Laval University/LOEX, Saint-Sacrement hospital Québec, QC, G1S 4L8, Canada; Department of Health Research Methods, Evidence and Impact, Faculty of Health Sciences, McMaster University, Hamilton, Ontario, L8S 4L8, Canada; CHU de Québec-Université Laval Research Centre, Endocrionology-nephrology, Population Health and Best Practices in Health and Immunology-infectiology axes, Québec, QC, G1V 4G2, Canada; Department of Medicine, Université Laval, Québec, QC, G1V 0A6, Canada; Institut National de Santé Publique du Québec, Evaluation of programs and surveillance of chronic diseases, injuries and their determinants, Statistical expertise and Information Centre, Québec, QC, G1V 5B3, Canada; Department of Preventive and Social Medicine, Université Laval, Québec, QC, G1V 0A6, Canada

**Keywords:** Paget’s disease of bone, mortality, endocrine diseases, cardiovascular diseases, administrative healthcare data, comorbidities, cardiovascular risk factors, material and social deprivation

## Abstract

Paget's disease of bone (PDB) is a focal, late-onset, chronic disorder of bone metabolism. Although the prevalence of PDB is stable in Quebec, the incidence and clinical severity of PDB have decreased over time. In this study, we determined the temporal trend of mortality associated with PDB in a large population-based retrospective cohort over 20 yr from the medico-administrative data of the *Régie de l'assurance maladie du Québec*, compared with the general population. For each fiscal year, the age-standardized rate of all-cause mortality was determined from 2000/2001 to 2020/2021 in individuals aged ≥55 with PDB and in the general population. The population size was 1 706 015 in 2000/2001 and 2 913 820 in 2019/2020. The case definition was based on 1 hospitalization or 2 claims from physicians with the diagnosis code of PDB. We determined the adjusted relative risk (aRR) of mortality in men and women, as well as by age categories. A total of 99% CIs have been calculated. We described the percentage of death for each main cause of death. Between 2000-2001 and 2020/2021, the all-cause age-standardized mortality rate (ASMR) in PDB individuals significantly increased from 47.1/1000 to 54.2/1000, respectively (JoinPoint analysis *p* < .01). The all-cause ASMR significantly decreased from 27.6/1000 to 22.3/1000 in the general population (JoinPoint analysis *p* < .01), respectively. Over the study period, compared with the general population, the PDB aRR of mortality rate has increased from 1.06 (0.95-1.19) to 1.16 (1.06-1.27), with a significant increase of mortality observed between 2016/2017 and 2019/2020. We observed an increase in the aRR of mortality in the age category of 65-74 yr from 1.15 (0.84-1.57) to 1.26 (0.97-1.64). The increased mortality in the endocrine and cardiovascular systems may be related to the aging of the pagetic cohort but also to the presence of vascular calcifications.

## Introduction

Paget's disease of bone (PDB) is a chronic late-onset disorder of bone metabolism. This focal increase in bone turnover may give rise to mechanical, vascular, and metabolic complications of this disease. In recent years, a declining prevalence, incidence, and clinical severity of PDB were reported in different countries.^1^^,^^2^ Although a stable prevalence of PDB was reported in the province of Quebec (Canada), the incidence and the clinical severity were reported to decrease over time.^3^^,^^4^ A decrease in mortality of patients with PDB was first reported in England between the years 1950 and 1970, with the highest rates of PDB-related mortality having been observed for cohorts born around 1880.^5^ However, in England and Wales, the mortality over 5 yr of follow-up reported in the General Practice Research Database (GPRD) was 33% in patients with PDB recruited between 1988 and 1999 (*n* = 2465) vs 28% in the control group (*n* = 7395), representing a relative risk of 1.4 (1.1-1.4).^6^ However, in the Olmsted County (Minnesota) population-based inception cohort, the standardized mortality ratios (SMRs) for all causes of death, in pagetic patients diagnosed between 1950 and 1994, were not significantly increased.^7^ Interestingly, in the context of declining incidence, prevalence, and clinical severity of PDB, no study on the evolution of mortality in PDB has been reported over the last 20 yr.

In pagetic patients from the GPRD, the most common causes of death were related to the circulatory system (38%), cancers (22%), and respiratory diseases (21%), with higher relative risks in patients with PDB than in controls, 1.5 (1.3-1.7), 1.8 (1.5-2.2), and 1.3 (1.1-1.6), respectively.^6^ The most frequent causes of death in the Olmsted County (Minnesota) population were related to the circulatory system (45%), cancers (19%), and respiratory diseases (12%), but, as opposed to the GPRD study,^6^ the causes of death SMR were not statistically different from controls.^7^ PDB is indeed associated with an increase in the incidence of cardiovascular diseases, in particular calcific valvular disease and generalized atherosclerosis. Aortic stenosis was reported in autopsy series in 24% of patients with PDB (*n* = 27) vs 4% of controls (*n* = 201) (*p* ˂ .01).^8^ In a radiographic study, 52% of patients with PDB (*n* = 42) had arteriosclerotic calcification vs 31% of controls (*n* = 36).^9^ Patients with PDB were reported to have more medial arterial calcification than in the control group, the latter being longer and thicker. Neoplastic complications may also occur in patients with PDB. The malignant transformation into pagetic sarcoma is reported in <0.3% of patients. In recent years, this transformation tends to be less frequent, although sarcoma can still be a PDB presenting feature.^10^ Pagetic sarcoma occurs in men with a mean age of 66 yr, predominating in the pelvis, with an average PDB duration of 15 yr.^11^ But patients with PDB may develop other malignancies, such as metastases as well as myeloma or lymphoma.^12^

In this study, we determined the temporal trend of all-cause mortality associated with PDB and causes of death in a large population-based retrospective cohort over 20 yr from the medico-administrative data of the *Régie de l'assurance maladie du Québec* (RAMQ) compared with the general population aged ≥55.

## Methods and methods

### Study design

The study design was a retrospective observational cohort study for each fiscal year from 2000/2001 to 2020/2021.

### Data sources and study population

Deaths occurring due to any cause were obtained from the Quebec Integrated Chronic Disease Surveillance System (QICDSS), the latter being housed at the National Institute of Public Health of Quebec (INSPQ).^13^ In this system, 5 healthcare administrative databases are linked for all residents in the province of Quebec, Canada, containing information related to the public healthcare insurance programs management, covering hospitalizations and fees for physician services. These databases rely on the health insurance registry (FIPA), hospital discharges (MedEcho), physician-billing claims (RAMQ), prescription drug claims, and the provincial death registry, as published.^14^ In 2020, according to demographic data from the Institut de la statistique du Quebec (https://statistique.quebec.ca/fr), ~34.2% of the population was 55 and over. The QICDSS was approved by Government bodies in legal possession of the databases and the public health ethics committee. The present study has been conducted as part of the ministerial plan of multi-thematic surveillance, which has received approval from the Public Health Ethic Committee (ISBN: 978-2-550-58 576-3).

### Study population

The case definition to identify individuals with PDB was based on 1 or more hospitalizations, or 2 or more claims from physicians with the PDB International classification of diseases (ICD) diagnosis code as follows: ICD9: 731.0, 731.1, 731.8, or 731.9; ICD10: M88.x. For each fiscal year of the study period, a PDB cohort and a general population cohort were created. The PDB cohort included all prevalent cases still alive on April, first of the year and all incident PDB cases meeting the case definition during the fiscal year. The general population cohort included all individuals aged ≥55 alive on April, first of the year and not included in the PDB cohort during the fiscal year.

### Statistical analysis

In the cohort with PDB, main characteristics were described during each fiscal year of the study period, including sex, age group, number of comorbidities (0-1, 2-4, $\ge$5), social and material deprivation index, and area of residence (Montreal census metropolitan area, others census metropolitan area, agglomeration, rural). Material and social deprivation indices are ecological proxies based on census data and postal codes.^15^ Undetermined material and social deprivation may have collective housing as their living environment or missing address. They are divided into quintiles, where quintile 1 includes the least deprived individuals and quintile 5 the most deprived. Material deprivation depends on education, income, and employment status. Social deprivation is determined by the proportion of people living alone, divorced, separated, or as single parents. We also described the most frequent comorbidities observed in the population with PDB as well as in the general population. A description of the general population for each fiscal year, including number of individuals, sex, age group, number of comorbidities (0-1, 2-4, $\ge$5), social and material deprivation index, and area of residence was also provided. Comorbidities were considered according to their number (0-1, 2-4, and 5+). A list of 31 diseases from the combined comorbidity index of Charlson and Elixhauser was used to count the number of comorbidities.^16^ Individuals were considered to have a comorbidity if there was 1 hospitalization or 2 claims in the fee-for-service medical services file separated by at least 30 d, with a comorbidity ICD diagnosis code, within a 5-yr period including the current year. For each fiscal year, between 2000/2001 and 2020/2021, the all-cause age-SMRs (ASMRs) in adults aged ≥55 with PDB and in the general population, were determined using the RAMQ data. The population size was706 015 in 2000/2001 and 913 820 in 2019/2020. For each fiscal year, the SMR ratio (SMRR) corresponds to the ratio of the ASMR in the Paget cohort to the ASMR in the general population. For each fiscal year from 2000/2001 to 2020/2021, robust Poisson regression models were also used to assess the adjusted relative risks (aRRs) of mortality among prevalent cases with PDB comparatively with individuals from the general population without PDB, adjusted for age, sex, material and social deprivation, comorbidities, and area of residence. All characteristics listed above were included as covariates. The ASMR and the aRR of mortality were also determined in men and women, as well as by age categories, namely 65-74 yr, 75-84 yr, and ≥ 85 yr for each fiscal year from 2000/2001 to 2020/2021. The age category 55-64 yr was not shown, as the number of deaths was very low in this category. The CIs were calculated at the 99% significance level. We described the percentage of death for each main cause of death up to 2019/2020, as data on causes of death were only provisional for the year 2020/2021. JoinPoint regression analyses were performed to analyze mortality trends in the group with PDB as well as in the general population. JoinPoint regression analyses were also performed on aRR to analyze trends over the years. Statistical analyses were performed using SAS version 7.1 (SAS Institute Inc.).

## Results

### Main characteristics of prevalent cases with PDB

Among the prevalent cases with PDB, between 2000/2001 and 2019/2020, we reported a decrease in the proportion of women from 54.2% to 47.7% (Table 1). Although the 85-yr-old and more age group have increased from 18.5% to 30.2% over the study period, the proportion of individuals in the 55-64 and 65-74 yr age group has decreased over time. Between 2000/2001 and 2019/2020, the percentage of pagetic patients with undetermined material and social deprivation has increased from 8% to 17.3%, reflecting mostly collective housing as a living environment. Between 2000/2001 and 2019/2020, the percentage of patients with PDB having 5 comorbidities and more has increased from 28.2% to 39.8%, whereas in the general population, which is younger than the cohort of prevalent cases with PDB, this proportion having ≥5 comorbidities has increased from 9.1% to 10.2% during this time period (Table 1).

**Table 1:** Main characteristics of prevalent cases with Paget’s disease of bone and the general population from 2000-2001 to 2019-2020 in the province of Quebec.

**Characteristics**	**Paget’s disease**					**General population**				
	**Fiscal year**					**Fiscal year**				
	**2000-2001**	**2005-2006**	**2010-2011**	**2015-2016**	**2019-2020**	**2000-2001**	**2005-2006**	**2010-2011**	**2015-2016**	**2019-2020**
**Number of cases, *N***	4925	6435	6725	7060	6990	1 706 015	1 971 820	2 288 435	2 639 190	2 906 635
**Women, %**	54.2	51.8	51.3	50.0	47.7	55.3	54.5	53.8	53.1	52.6
**Age groups, %**										
**55-64 yr**	13.7	12.7	10.1	9.1	9.3	43.7	46.5	46.3	44.5	42.5
**65-74 yr**	31.5	29.2	27.4	27.0	24.9	32.1	28.8	29.2	31.6	32.6
**75-84 yr**	36.3	37.4	37.7	35.7	35.6	18.5	18.7	17.7	16.6	17.4
**85+ years**	18.5	20.7	24.8	28.2	30.2	5.8	6.0	6.8	7.2	7.5
**Material deprivation, %**										
**1 (most privileged)**	15.3	16.1	14.8	13.0	13.5	18.0	17.8	17.2	17.2	17.0
**2**	18.0	17.8	16.2	14.3	15.0	17.7	17.8	17.7	17.1	17.5
**3**	19.9	18.4	18.2	17.4	16.6	19.5	19.6	18.4	18.8	18.6
**4**	19.9	19.4	18.8	18.8	19.6	20.1	19.7	19.7	19.0	19.5
**5 (most deprived)**	19.0	18.9	17.7	18.4	19.1	20.3	19.8	19.3	19.4	19.3
**Undetermined**^**a**^	7.8	9.4	14.4	18.2	16.3	4.4	5.3	7.7	8.5	8.0
**Social deprivation, %**										
**1 (most privileged)**	13.5	13.5	13.0	14.1	13.8	16.4	17.2	17.2	18.0	18.3
**2**	16.6	15.3	15.4	15.6	16.8	17.8	18.0	17.8	18.7	19.3
**3**	17.2	17.9	17.1	16.1	16.6	19.5	19.2	18.9	18.3	18.6
**4**	21.7	20.4	18.8	17.1	17.0	21.2	20.2	19.2	18.5	18.3
**5 (most deprived)**	23.2	23.5	21.3	18.9	19.7	20.8	20.2	19.2	18.0	17.6
**Undetermined**^**a**^	7.8	9.4	14.4	18.2	16.3	4.4	5.3	7.7	8.5	8.0
**Number of comorbidities, %**										
**0-1**	32.4	31.0	30.7	29.0	30.3	61.8	61.1	62.9	64.8	67.4
**2-4**	39.4	36.8	35.6	32.9	29.9	29.1	29.0	27.5	25.0	22.4
**≥5**	28.2	32.2	33.6	38.1	39.8	9.1	9.9	9.6	10.2	10.2
**Area of residence, %**										
** Montreal census metropolitan area**	48.2	46.3	44.6	40.3	39.6	45.5	45.2	44.3	45.1	45.1
** Others census metropolitan area**	19.0	21.0	21.5	24.5	26.3	18.3	19.5	20.2	20.5	21.8
** Agglomerations**	13.1	13.8	14.4	14.3	13.8	12.6	13.1	13.3	12.1	11.5
** Rural**	19.1	18.5	19.2	20.5	20.1	22.8	21.8	21.8	21.9	21.3
** Unknown**	0.6	0.3	0.3	0.4	0.2	0.9	0.5	0.4	0.4	0.3

a A large proportion of those with undetermined material and social deprivation may have collective housing as their living environment or missing address.

Hypertension, diabetes, and obesity were twice more frequent in PDB than in the general population (Table 2). All causes of cardiovascular conditions were also more frequently reported in pagetic patients than in the general population.

**Table 2:** Details of main comorbidities observed in patients with Paget’s disease of bone and in the general population.

**Comorbidities**	**Paget’s disease**					**General population**				
	**Fiscal year**					**Fiscal year**				
	**2000-2001**	**2005-2006**	**2010-2011**	**2015-2016**	**2019-2020**	**2000-2001**	**2005-2006**	**2010-2011**	**2015-2016**	**2019-2020**
**Cardiovascular risk factor, %**										
**Hypertension**	53.4	62.0	62.7	62.1	59.2	37.3	41.9	39.3	34.6	29.4
**Diabetes**	18.7	23.8	27.4	31.2	32.4	12.2	14.5	16.2	16.1	15.0
**Obesity**	4.2	5.7	6.1	7.4	8.5	2.7	3.4	3.1	3.3	3.3
**Cardiovascular disease, %**										
**Cerebrovascular disease**	14.4	13.5	11.4	11.4	11.2	6.3	5.2	4.1	3.8	3.6
**Congestive heart failure**	15.1	13.7	13.1	14.8	15.4	5.1	4.3	3.8	3.7	3.8
**Myocardial infarction**	12.0	12.5	10.6	12.9	13.3	5.3	5.0	4.0	4.0	4.0
**Cardiac arrhythmias**	19.7	21.1	23.3	25.7	27.2	8.7	8.9	8.9	9.5	9.4
**Valvular heart disease**	8.2	9.1	9.6	11.5	12.9	3.2	3.3	3.1	3.4	3.6
**Peripheral vascular disorder**	12.8	14.1	15.8	17.8	20.8	5.3	4.5	4.5	4.4	4.3
**Other conditions, %**										
**Dementia**	9.3	11.1	13.3	13.8	14.9	2.6	3.2	3.6	3.7	3.7
**Cancer, with and without metastasis**	20.1	21.0	22.4	24.8	24.7	9.7	10.6	11.8	12.6	12.9
**Depression**	11.0	11.2	9.7	9.7	9.1	7.6	7.8	7.5	7.2	6.2
**Neurological disorders**	6.8	7.1	7.7	8.1	8.2	2.8	2.8	2.9	3.1	3.1
**Rheumatoid arthritis**	5.3	5.5	5.3	6.2	6.9	2.2	2.2	2.3	2.6	2.8
**Chronic pulmonary disorder**	27.8	24.5	23.1	21.5	21.8	16.1	13.3	11.6	10.9	10.7

### Age-standardized mortality rate in PDB and in the general population

**Figure 1:**
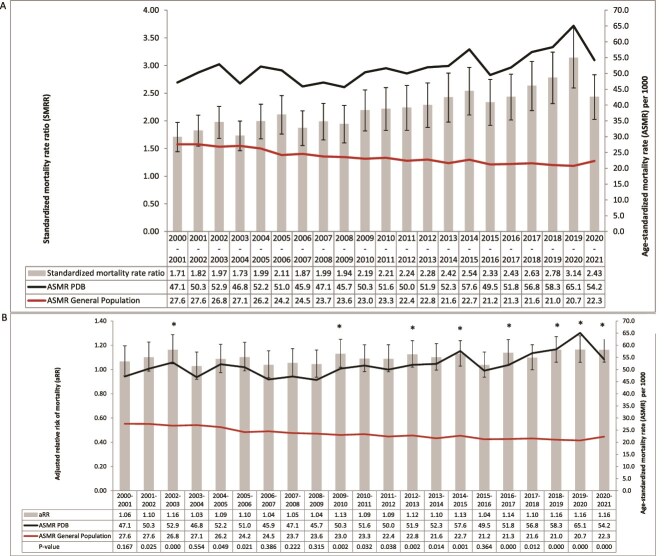
Age-standardized^#^ mortality rate and (A) standardized mortality rate ratio; (B) adjusted relative risk of mortality among individuals aged 55 and over with Paget's disease of bone in Quebec, from 2000-2001 to 2020-2021. ^#^Rate adjusted based on the age structure of the Quebec population in 2011. Footnote: statistically significant difference (^*^p < .01).

**Figure 2:**
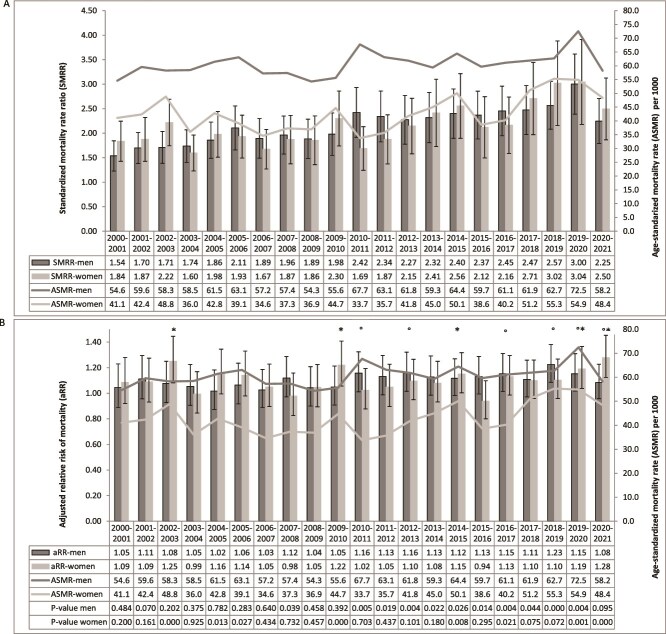
Age-standardized^#^ mortality rate and (A) standardized mortality rate ratio; (B) adjusted relative risk of mortality among women and men aged 55 and over with Paget’s disease of bone in Québec, from 2000-2001 to 2020-2021. ^#^Rate adjusted from the age structure of the Quebec population in 2001. Footnote: statistically significant difference (*p* < .01) in women (^*^) or in men (°).

Between 2000/2001 and 2020/2021, the all-cause ASMRs in pagetic patients aged ≥55 increased from 47.1/1000 to 54.2/1000 (Figure 1). The increase in all-cause ASMRs in PDB was observed in both men and women (Figure 2A). In both men and women with PDB, the JoinPoint regression analyses showed that the average annual percentage change from 2001 to 2021 (95% CI) was 0.9 (0.4, 1.4) *p* < .01, this change being 1.0 (0.0, 2.0) *p* = .1 in the subgroup of women with PDB, and 0.5 (0.1, 1.0) *p* < .01 in men. In the general population, the all-cause ASMRs decreased from 27.6/1000 in 2000/2001 to 22.3/1000 in 2020/2021. In both men and women from the general population, the JointPoint regression analyses showed that the average annual percentage change from 2001 to 2021 (95% CI) was −1.4 (−1.8, −1.0) *p* < .01, this change being −1.1 (−1.5, −0.7) *p* < .01 in the subgroup of women, and −1.7 (−2.2, −1.2) *p* < .01 in men.

### Standardized mortality rate ratio in individuals with PDB according to age categories

**Figure 3:**
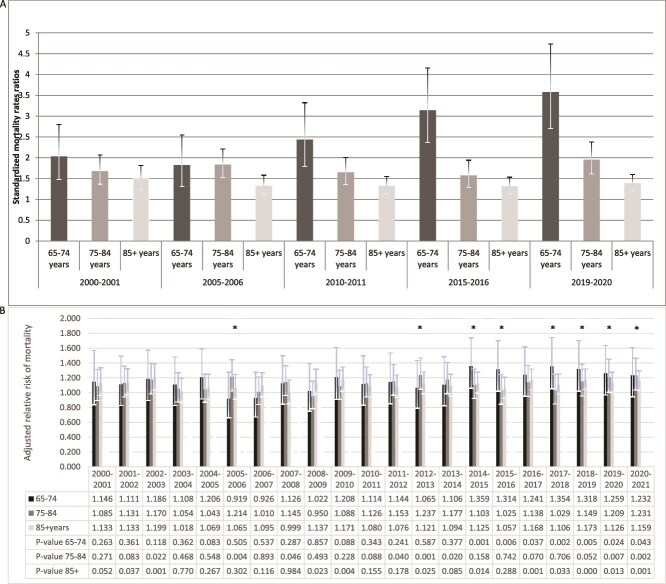
(A) Standardized mortality rate ratio and (B) adjusted relative risk of mortality among individuals aged 65 and over with Paget’s disease of bone in Québec, from 2000-2001 to 2020-2021. Footnote: statistically significant difference (^*^p < .01).

The highest increase in SMRR has been observed in pagetic patients in the age category of 65-74 yr, from 2.03 (1.48-2.80) in 2000/2001 to 3.58 (2.70-4.73) in 2019/2020 (Figure 3A).

### Adjusted relative risk of mortality

The death rate was found to increase from years 2016/2017 to 2019/2020 according to visual examination of the 99% CIs on the aRRs in Figure 1B, where we observe a significant increase in mortality. Indeed, the JoinPoint model identified a significant breakpoint in the trend of aRR in 2015-2016. Between years 2000 and 2015, the average annual aRR was 1.086 (1.057-1.116) (*p* < .0001) vs an average annual aRR of 1.146 (1.099-1.195) between 2016 and 2020 (*p* < .0001). The aRR of mortality in prevalent cases of PDB comparatively with individuals from general population without PDB has increased from 1.06 (0.95-1.19) in 2000/2001 to 1.16 (1.06-1.27) in 2019/2020, with a significant increase of mortality observed between 2016/2017 and 2019/2020 (Table 3, Figure 1B) and particularly in men from 1.15 (1.01-1.31) in 2016/2017 to 1.15 (1.02-1.31) in 2019/2020 (Figure 2B). An increase in the aRR of mortality was observed between 2015/2016 and 2019/2020 for diseases of the endocrine system from 1.44 (0.86-2.42) to 2.43 (1.58-3.72), for cardiovascular diseases from 1.03 (0.84-1.27) to 1.28 (1.06-1.55) (Table 4), while no increase was observed for nervous system diseases, cancer, and respiratory diseases. We observed an increase in the aRR of mortality in the age category of 65-74 yr from 1.15 (0.84-1.57) to 1.26 (0.97-1.64) as well as in the category of 75-84 yr, from 1.08 (0.90-1.31) to 1.21 (1.01-1.45) (Figure 3B).

**Table 3:** All-cause age-standardized mortality rates, standardized mortality rate ratios, and adjusted relative risks of mortality from 2000-2001 to 2019-2020 in the province of Quebec.

**Characteristics**	**Fiscal year**				
	**2000-2001**	**2005-2006**	**2010-2011**	**2015-2016**	**2019-2020**
**All-cause age-standardized mortality rates /1000 (CI 99%)**					
** Paget cases**	47.1 (39.6-56.3)	51.0 (42.3-61.3)	51.6 (42.3-62.9)	49.5 (40.4-60.6)	65.1 (53.4-78.9)
** General population (GP)**	27.6 (27.3-27.9)	24.2 (23.9-24.5)	23.3 (23.1-23.6)	21.2 (21.0-21.4)	20.7 (20.5-20.9)
**Standardized mortality rate ratios (CI 99%)**					
** SMRRs**	1.71 (1.44-2.02)	2.11 (1.76-2.52)	2.21 (1.82-2.68)	2.33 (1.92-2.84)	3.14 (2.59-3.79)
**Adjusted relative risks (aRR) of mortality (CI 99%)** ^**a**^					
** aRR**	1.06 (0.95-1.19)	1.10 (0.99-1.22)	1.09 (0.98-1.20)	1.04 (0.94-1.15)	1.16 (1.06-1.27)
**All-cause age-standardized mortality rates /1000 by sex (CI 99%)**					
** Paget Women**	41.1 (31.4-54.2)	39.1 (29.8-51.6)	33.7 (24.3-47.3)	38.6 (26.5-55.8)	54.9 (38.3-77.6)
** GP Women**	22.4 (22.0-22.7)	20.3 (19.9-20.6)	20.0 (19.7-20.3)	18.2 (17.9-18.5)	18.0 (17.7-18.3)
** Paget Men**	54.6 (43.2-69.5)	63.1 (49.0-80.6)	67.7 (52.9-86.2)	59.7 (46.9-75.9)	72.5 (57.0-91.5)
** GP Men**	35.5 (34.9-36.2)	30.0 (29.4-30.5)	27.9 (27.5-28.4)	25.1 (24.7-25.5)	24.1 (23.8-24.5)
**Standardized mortality rate ratios by sex (CI 99%)**					
** Women**	1.84 (1.43-2.36)	1.93 (1.49-2.50)	1.69 (1.24-2.29)	2.12 (1.50-3.00)	3.04 (2.18-4.26)
** Men**	1.54 (1.23-1.92)	2.11 (1.66-2.68)	2.42 (1.92-3.07)	2.37 (1.88-2.98)	3.00 (2.39-3.77)
**Adjusted relative risks (aRR) of mortality by sex (CI 99%)** ^**a**^					
** Women**	1.08 (0.92-1.28)	1.14 (0.98-1.33)	1.02 (0.88-1.19)	0.94 (0.80-1.10)	1.19 (1.04-1.37)
** Men**	1.04 (0.89-1.23)	1.06 (0.92-1.24)	1.16 (1.01-1.33)	1.13 (0.99-1.29)	1.15 (0.86-1.23)
**All-cause age-standardized mortality rates /1000 by age groups (CI 99%)**					
** Paget 65-74 yr**	41.8 (29.6-57.1)	31.9 (22.3-44.1)	37.9 (27.3-51.3)	44.6 (33.1-58.7)	48.8 (36.3-64.2)
** GP 65-74 yr**	20.6 (20.1-21.1)	17.5 (17.0-17.9)	15.5 (15.2-15.9)	14.2 (13.8-14.5)	13.6 (13.3-13.9)
** Paget 75-84 yr**	86.6 (69.7-106.2)	81.1 (66.9-97.3)	71.1 (58.2-86.0)	61.6 (49.6-75.6)	70.3 (57.3-85.2)
** GP 75-84 yr**	51.6 (50.5-52.6)	44.1 (43.3-45.1)	43.1 (42.3-44.0)	39.0 (38.2-39.8)	35.9 (35.2-36.6)
** Paget 85+ years**	203.3 (166.9-245.1)	165.4 (138.1-196.4)	167.7 (143.0-195.2)	150.7 (129.3-174.7)	168.2 (146.1-192.6)
** GP 85+ years**	135.9 (132.9-139.0)	124.7 (122.0-127.4)	126.3 (124.0-128.6)	114.2 (112.2-116.2)	120.0 (119.0-122.9)
**Standardized mortality rate ratios by age groups (CI 99%)**					
** 65-74 yr**	2.03 (1.48-2.80)	1.83 (1.3/−2.55)	2.44 (1.79-3.32)	3.14 (2.37-4.15)	3.58 (2.70-4.73)
** 75-84 yr**	1.68 (1.36-2.07)	1.84 (1.53-2.21)	1.65 (1.36-2.00)	1.58 (1.28-1.94)	1.96 (1.61-2.38)
** 85+ years**	1.49 (1.23-1.81)	1.33 (1.11-1.58)	1.33 (1.14-1.55)	1.32 (1.14-1.53)	1.39 (1.21-1.60)
**Adjusted relative risks (aRR) of mortality by age groups (CI 99%)** ^**a**^					
** 65-74 yr**	1.15 (0.84-1.57)	0.92 (0.66-1.27)	1.11 (0.83-1.49)	1.31 (1.02-1.70)	1.26 (0.97-1.64)
** 75-84 yr**	1.08 (0.90-1.31)	1.21 (1.02-1.44)	1.13 (0.94-1.35)	1.02 (0.84-1.25)	1.21 (1.01-1.45)
** 85+ years**	1.13 (0.96-1.34)	1.06 (0.91-1.24)	1.08 (0.94-1.24)	1.06 (0.92-1.21)	1.13 (0.99-1.27)

a Adjusted relative risks of all-cause mortality in Paget cases vs General population estimated from a Robust Poisson regression model (adjusted for age, sex, number of comorbidities (0-1, 2-4, 5+), material and social deprivation, and area of residence).

**Table 4:** Age-standardized mortality rates /1000, standardized mortality rate ratios, and adjusted relative risks of mortality from 2000-2001 to 2019-2020 in the province of Quebec by specific cause.

**Characteristics**	**Fiscal year**				
	**2000-2001**	**2005-2006**	**2010-2011**	**2015-2016**	**2019-2020**
**All-cause age-standardized mortality rates /1000 (CI 99%) Cancer**					
**Paget cases**	14.3 (9.8-20.7)	12.3 (8.4-17.8)	11.9 (7.8-18.1)	14.7 (9.5-22.2)	14.7 (9.3-22.5)
**General population**	6.5 (6.4-6.7)	6.1 (6.0-6.3)	5.9 (5.8-6.0)	5.5 (5.4-5.6)	5.2 (5.1-5.3)
**SMRRs (CI 99%)**	2.19 (1.55-3.10)	2.00 (1.41-2.84)	2.01 (1.36-2.98)	2.65 (1.78-3.94)	2.83 (1.87-4.28)
**aRR**^**a**^ **(CI 99%)**	1.22 (0.98-1.53)	1.04 (0.84-1.29)	0.98 (0.80-1.22)	1.04 (0.85-1.27)	0.95 (0.77-1.17)
**All-cause age-standardized mortality rates /1000 (CI 99%) Cardiovascular diseases**					
**Paget cases**	10.7 (8.0-15.1)	14.7 (9.8-21.4)	11.4 (7.3-17.5)	11.1 (6.9-17.6)	13.3 (8.5-20.4)
**General population**	7.2 (7.0-7.3)	5.6 (5.4-5.7)	4.9 (4.8-5.0)	4.3 (4.2-4.4)	4.0 (3.9-4.1)
**SMRRs (CI 99%)**	1.50 (1.14-1.97)	2.63 (1.82-3.79)	2.32 (1.54-3.48)	2.59 (1.68-4.00)	3.33 (2.21-5.01)
**aRR**^**a**^ **(CI 99%)**	0.95 (0.77-1.19)	1.08 (0.88-1.34)	1.08 (0.87-1.32)	1.03 (0.84-1.27)	1.28 (1.06-1.55)
**All-cause age-standardized mortality rates /1000 (CI 99%) Respiratory diseases**					
**Paget cases**	3.3 (2.1-6.5) 1.8 (1.7-1.9)	3.9 (2.3-7.2)	5.7 (3.0-10.7)	2.4 (1.6-5.6)	5.3 (2.5-10.5)
**General population**		1.7 (1.7-1.8)	1.9 (1.8-2.0)	1.6 (1.6-1.7)	1.8 (1.7-1.8)
**SMRRs (CI 99%)**	1.80 (1.19-2.72)	2.22 (1.37-3.60)	2.97 (1.67-5.28)	1.46 (0.99-2.16)	2.99 (1.58-5.66)
**aRR**^**a**^ **(CI 99%)**	1.11 (0.74-1.66)	1.24 (0.88-1.75)	1.26 (0.93-1.70)	0.83 (0.57-1.19)	0.95 (0.69-1.32)
**All-cause age-standardized mortality rates /1000 (CI 99%) Diseases of the endocrine system**					
**Paget cases**	2.10 (0.64-6.16)	4.49 (1.83-9.49)	3.49 (1.14-8.65)	1.92 (0.52-6.11)	5.86 (2.30-12.5)
**General population**	0.90 (0.85-0.96)	0.82 (0.77-0.87)	0.72 (0.68-0.76)	0.51 (0.48-0.55)	0.45 (0.42-0.48)
**SMRRs (CI 99%)**	2.33 (0.91-6.00)	5.48 (2.58-11.6)	4.86 (1.97-12.0)	3.73 (1.34-10.4)	13.1 (6.01-28.6)
**aRR**^**a**^ **(CI 99%)**	1.08 (0.59-1.99)	1.62 (1.02-2.56)	1.40 (0.86-2.28)	1.44 (0.86-2.42)	2.43 (1.58-3.72)
**All-cause age-standardized mortality rates /1000 (CI 99%) Diseases of the nervous systems**					
**Paget cases**	1.24 (0.56-4.43)	1.36 (0.73-3.95)	2.15 (1.37-5.18)	1.16 (0.66-4.38)	3.22 (1.16-7.95)
**General population**	1.22 (1.15-1.28)	1.26 (1.20-1.32)	1.28 (1.22-1.34)	1.18 (1.13-1.23)	1.08 (1.04-1.13)
**SMRRs (CI 99%)**	1.01 (0.52-1.99)	1.08 (0.62-1.86)	1.68 (1.11-2.53)	0.98 (0.59-1.63)	2.97 (1.28-6.87)
**aRR**^**a**^ **(CI 99%)**	0.78 (0.40-1.52)	0.86 (0.50-1.48)	1.33 (0.90-1.98)	0.81 (0.50-1.31)	1.37 (0.94-2.01)

a Adjusted relative risks (aRRs) of all-cause mortality in patients with Paget’s disease vs general population estimated from a Robust Poisson regression model (adjusted for age, sex, number of comorbidities (0-1, 2-4, 5+), material and social deprivation, and area of residence).

### Causes of death by systems in individuals with PDB

Between 2000/2001 and 2019/2020, the absolute number of deaths in pagetic patients aged ≥55 varied from 410 to 655. In 2019/2020, the most frequent causes of death in pagetic patients were cardiovascular diseases (28%), cancer (23%), respiratory diseases (10%), and nervous system diseases (7%) (Table 5). The death by cancer in patients with PDB varied from 32% in 2000/2001% to 23% in 2019/2020. The death by cardiovascular diseases in patients with PDB has varied from 32% in 2000/2001% to 28% in 2019/2020. Although the death caused by respiratory diseases remained stable over 20 yr, the death by nervous system diseases increased from 4% in 2000/2001% to 7% in 2019/2020.

**Table 5:** Main causes of mortality by systems in cases with Paget’s disease from 2000-2001 to 2019-2020 in the province of Quebec.

**Characteristics**	**Fiscal year**				
	**2000-2001**	**2005-2006**	**2010-2011**	**2015-2016**	**2019-2020**
**Number of deaths in cases with Paget’s disease**	410	500	555	570	655
**Main causes of mortality, %**					
**Cancer**	32.0	28.9	25.4	29.7	23.3
**Cardiovascular diseases**	32.0	29.9	27.3	27.0	27.9
**Digestive diseases**	2.6	3.1	2.7	3.6	3.9
**Infectious diseases**	1.3	3.1	3.6	1.8	3.1
**Diseases of musculoskeletal system**	1.3	2.1	0.9	2.7	1.6
**Respiratory diseases**	10.3	11.3	13.6	9.0	10.1
**Diseases states**	0	0	0.9	0.9	0.8
**Organic syndromes**	3.8	4.1	5.4	6.3	7.0
**Diseases of endocrine system**	5.1	6.2	5.4	4.5	5.4
**Diseases of the nervous systems**	3.8	5.1	8.2	5.4	7.0
**Trauma**	2.6	2.1	1.8	3.6	5.4
**Mental disorders**	0	0	0	0.9	0
**Others**	5.1	4.1	4.5	4.5	4.6

### System-specific causes of death in individuals with PDB (2000-2020)

Regarding cardiovascular diseases, the main specific causes of death in patients with PDB were ischemic heart disease (including myocardial infarction) in 50.6%, cerebrovascular diseases 17.1%, heart failure 10.6%, vascular diseases 6.1%, peripheral vascular diseases 4.5%, cardiac arrhythmias (including cardiac arrest) 6.3%, and other circulatory system diseases 4.8%. In the nervous system, the main causes of death were dementia (including Alzheimer's) in 60.9%, Parkinson's 18.0%, epilepsy 2.3%, multiple sclerosis 2.3%, and other neurological diseases 2.3%. In the endocrine system, the main causes of death were diabetes in 76.2%, obesity in 2.5%, malnutrition 4.9%, thyroid gland disorders 4.1%, and other metabolic abnormalities 12.3%. Among respiratory diseases, the main causes of death were chronic obstructive pulmonary disease in 57.2%, pneumonia, and acute bronchitis 30.9%, respiratory diseases affecting interstitial tissue 8.6%, and other respiratory diseases 3.3%.

### Trends of the standardized mortality rate ratio over time and by system-specific causes of death

The SMRR has increased from 1.71 (1.44-2.02) in 2000-2001 to 3.14 (2.59-3.79) in 2019-2020 (Table 3), particularly in men from 1.54 (1.23-1.92) in 2000-2001 to 3.00 (2.39-3.77). Between 2015/2016 and 2019/2020, we observed an increase in the SMRR for endocrine system diseases, from 3.73 (1.34-10.4) to 13.1 (6.01-28.6), as well as for the nervous system diseases, which increased from 0.98 (0.59-1.63) to 2.97 (1.28-6.87) (Table 4). We reported a progressive increase of the SMRR for the diseases of the cardiovascular system, the latter having more than doubled over a 20 yr-period, up to 3.33 (2.21-5.01) in 2019-2020, while this increase is more moderate for cancer and respiratory diseases.

## Discussion

In this study, between 2000/2001 and 2020/2021, the all-cause ASMRs in pagetic patients aged ≥55 increased significantly from 47.1/1000 to 54.1/1000, the latter having decreased from 27.6/1000 to 22.3/1000 in the general population during this time period. We reported an increase in the absolute number of deaths in pagetic patients aged ≥55 from 410 to 655 over 20 yr. We observed an increase in aRR of mortality in PDB in both men and women, with a significant increase of mortality observed between 2016/2017 and 2019/2020. The most common causes of death in patients with PDB were endocrine system diseases and cardiovascular diseases. We observed an increase in the aRR of mortality in the age category of 65-74 yr as well as in the category of 75-84 yr. Although the number of patients in this age category 65-74 yr was lower than in the other age categories, due to the usual late onset of PDB, this information may suggest an early onset of PDB, possibly related to a familial form and/or more severe phenotype of PDB.

The increase in ASMR in PDB reported in this study may be related to the aging of the pagetic cohort. The difference in mortality rates according to SMRR, which is adjusted for age effect, and aRR, which is adjusted for age, sex, material and social deprivation, and area of residence as well as cumulative diseases related to aging, suggest that the increasing number of comorbidities over time may explain the frailty observed in the population with PDB. The higher proportion of individuals of undetermined status for material and social deprivation may suggest collective housing, indeed supporting the aging of the PDB cohort. The leading cause of death in pagetic patients in the endocrine and cardiovascular diseases is interesting. Indeed, increased cardiovascular morbidity and mortality in PDB have already been reported in the literature.^6^^,^^7^^,^^17^ In a recent pilot study of 12 patients with PDB and 58 controls, the aortic pulse wave velocity was significantly higher in pagetic patients than in controls, whereas the carotid artery intima media thickness was not significantly increased.^18^ The higher prevalence of altered glucose tolerance and hypertension previously reported in PDB may contribute to this increased cardiovascular mortality.^19^ Furthermore, high serum calcium and/or increased bone alkaline phosphatase in PDB may explain the link between PDB and cardio-metabolic disorders.^19^ In a pilot study of 23 patients with PDB and 30 controls, a correlation between alkaline phosphatase level and hypertension medication has indeed been reported.^20^ In a recent study comparing the clinical severity of PDB in a contemporary cohort with a historical cohort in Quebec, atherosclerotic coronary disease was observed in 18% of patients from the contemporary cohort vs 21% in the historical cohort.^3^ Furthermore, 57% of pagetic patients of the contemporary cohort had hypertension vs 32% in the historical cohort but these changes were not statistically significant. Differences in comorbidity occurrence may be related to differences in lifestyle habits. For instance, tobacco smoke was reported to be associated with PDB in a French study of families with PDB^21^ but this association has not been further reported in other populations, including our French-Canadian cohort.^22^ A possible protective effect of zoledronic acid use in pagetic patients against cancer could also be hypothesized.^23^^,^^24^ The contribution of the strong genetic component of PDB, mainly the mutations in the *SQSTM1* gene and their impact on cell proliferation, signaling, and function, on the causes of mortality observed in patients with PDB is yet unknown, although a decreased level of the SQSTM/p62 protein was associated with vascular calcifications.^25^^,^^26^ Some *SQSTM1* gene mutations linked to PDB were reported in chronic nervous system disorders, such as frontotemporal dementia and amyotrophic lateral sclerosis,^27^ suggesting that both PDB and nervous system diseases may coexist.

This study provides for the first time some Canadian epidemiological data on mortality in PDB. The studied sample size is 2 914 480 individuals in 2019/2020, all aged ≥55 yr, this sample represents 37% of inhabitants in Quebec and 98% of Quebec inhabitants aged ≥55 yr, as well as the quality of the studied data represents some strengths of this study.

This study also has some limitations. For instance, we did not verify the billing codes in medical records. According to our PDB case definition, we have only studied patients aged ≥55 with at least 1 hospitalization or 2 claims from physicians with the PDB diagnosis code. In this study, 37% of incident cases with PDB were identified through hospitalization. Between 2000/2001 and 2020/2021, 56.4% of the identified cases met again this PDB case definition at least 1 more time, 81% within 2 yr and 19% more than 2 yr after their first identification. Using this PDB case definition, we may have underestimated the true mortality rate of PDB in Quebec, as only one-third of pagetic patients are symptomatic. Considering this high frequency of asymptomatic PDB, the current decline in PDB clinical severity and the most recent guidelines recommending bisphosphonates prescription to mainly control pagetic bone pain,^28^^,^^29^ we did not consider zoledronic acid or risedronate prescription in our PDB case definition, the latter having possibly decreased the specificity of our PDB case definition.

Our results support the clinical utility of an epidemiological surveillance program of PDB in Quebec. Indeed, the higher mortality rate in patients with PDB in Quebec would require further studies, as it may give rise to specific recommendations for clinical management, prevention, and long-term follow-up of these patients.

## Conclusion

We reported an increase in mortality from the endocrine and cardiovascular systems, possibly related to the aging of the pagetic cohort resulting in an accumulation of comorbidities over time. The presence of vascular calcifications, well known in PDB, possibly related to the SQSTM1 role in autophagy, may also play a role. This increase in cardiovascular mortality in pagetic patients over time is of interest, as screening and early preventive measures on comorbidities could be considered for patients with PDB.

## Data Availability

Data will be made available on request.
